# Redetermination of MoPt_3_Si_4_ from single-crystal data

**DOI:** 10.1107/S1600536810054425

**Published:** 2011-01-22

**Authors:** Stephane Knittel, Michel Francois, Jean Steinmetz, Michel Vilasi

**Affiliations:** a Institut Jean Lamour, UMR 7198, Département 2, Université Henri Poincaré, Nancy Université, Faculté des Sciences et Techniques, BP 239, 54506 Vandoeuvre les Nancy CEDEX, France

## Abstract

The crystal structure of molybdenum triplatinum tetrasilicide, MoPt_3_Si_4_, determined previously from powder diffraction data [Joubert *et al.* (2010 ▶). *J. Solid State Chem.* **183**, 173–179], has been redetermined using a single crystal synthesized from the elements by high-frequency melting. The redetermination provides more precise geometrical data and also anisotropic displacement parameters. The crystal structure can be considered to be derived from the PtSi structure type with an ordered substitution of Pt by Mo atoms, but leading to a very distorted Si network compared to the parent structure. Mo and Pt exhibit different coordination polyhedra. These are based on bicapped-square anti­prisms, but with two additional vertices in *cis* positions for Mo, whereas they are in *trans* positions for Pt (as in PtSi). The coordination polyhedra for three of the Si atoms can be considered as highly deformed square anti­prisms (as in PtSi), while the fourth Si atom has a bicapped trigonal–prismatic coordination geometry.

## Related literature

For general background to molybdenum silicides, see: Littner (2003 ▶); Benarchid *et al.* (2009 ▶); Bernard *et al.* (2010 ▶); Cabouro *et al.* (2007 ▶, 2008 ▶); Fitzer (1955 ▶); Knittel *et al.* (2010 ▶). For the structure determination of the title compound from standard X-ray powder diffraction data, see: Joubert *et al.* (2010 ▶). For the PAP correction program, see: Pouchou & Pichoir (1984 ▶).

## Experimental

### 

#### Crystal data

MoPt_3_Si_4_
*M* *_r_* = 793.57
Orthorhombic, 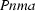

*a* = 5.5121 (2) Å
*b* = 3.4951 (1) Å
*c* = 24.3078 (7) Å
*V* = 468.30 (3) Å^3^
*Z* = 4
Mo *K*α radiation
μ = 92.80 mm^−1^
*T* = 297 K
0.12 × 0.03 × 0.03 mm

#### Data collection

Bruker APEXII QUAZAR CCD diffractometer
Absorption correction: multi-scan (*SADABS*; Bruker, 2004 ▶) *T*_min_ = 0.031, *T*_max_ = 0.174
9779 measured reflections
1060 independent reflections
955 reflections with *I* > 2σ(*I*)
*R*_int_ = 0.040

#### Refinement

*R*[*F* ^2^ > 2σ(*F* ^2^)] = 0.029
*wR*(*F* ^2^) = 0.067
*S* = 1.41
1060 reflections
50 parameters
Δρ_max_ = 2.16 e Å^−3^
Δρ_min_ = −3.81 e Å^−3^

### 

Data collection: *APEX2* (Bruker, 2004 ▶); cell refinement: *SAINT* (Bruker, 2004 ▶); data reduction: *SAINT*; program(s) used to solve structure: *SHELXS97* (Sheldrick, 2008 ▶); program(s) used to refine structure: *SHELXL97* (Sheldrick, 2008 ▶); molecular graphics: *DIAMOND* (Brandenburg, 1999 ▶) and *ORTEP-3* (Farrugia, 1997 ▶); software used to prepare material for publication: *SHELXL97*.

## Supplementary Material

Crystal structure: contains datablocks global, I. DOI: 10.1107/S1600536810054425/fi2101sup1.cif

Structure factors: contains datablocks I. DOI: 10.1107/S1600536810054425/fi2101Isup2.hkl

Additional supplementary materials:  crystallographic information; 3D view; checkCIF report

## Figures and Tables

**Table 1:** Selected bond lengths (Å)

Pt1—Si3^i^	2.406 (3)
Pt1—Pt1^ii^	2.9119 (6)
Pt2—Si1^iii^	2.387 (3)
Pt2—Mo1^iv^	2.9331 (10)
Pt3—Si3	2.405 (2)
Pt3—Pt2	2.8874 (5)
Mo1—Si4	2.535 (2)
Mo1—Pt2^iii^	2.9331 (10)
Si1—Pt2^iv^	2.387 (3)
Si1—Si2	2.711 (3)
Si2—Pt3	2.466 (3)
Si2—Si1	2.711 (3)
Si3—Pt1^v^	2.590 (2)
Si3—Si3^vi^	2.807 (5)
Si4—Pt2^v^	2.407 (2)
Si4—Si4^vii^	3.012 (5)

Symmetry codes: (i) 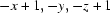; (ii) 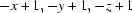; (iii) 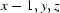; (iv) 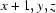; (v) 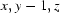; (vi) 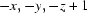; (vii) 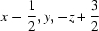.

**Fig. 1.**
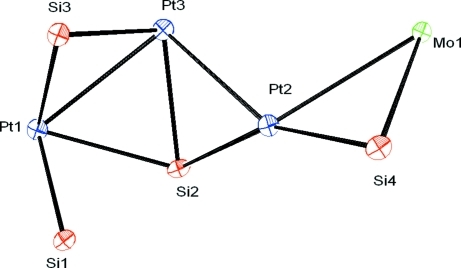
Plot of the asymmetric unit of MoPt3Si4. The displacement ellipsoids are drawn at the 75% probability level. Atoms are bonded for interatomic distances lower than 3.1 Å.

**Fig. 2.**
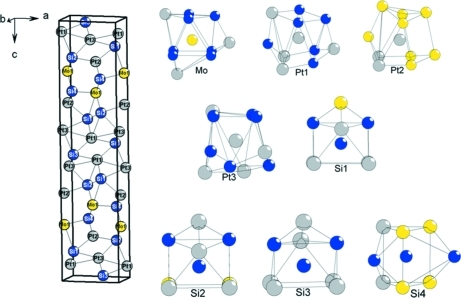
The unit cell and the coordination polyhedra of the atoms in the structure of MoPt3Si4. Si in blue, Pt in light grey, Mo in yellow.

**Fig. 3.**
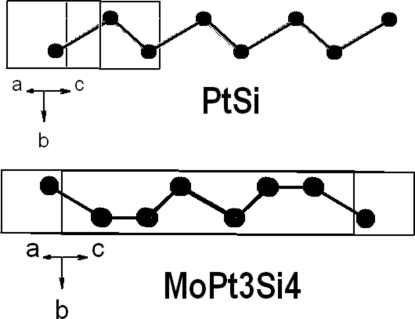
Corrugated ribbons from Si atoms in the structure of PtSi and MoPt3Si4.

## supplementary crystallographic information

### Comment

Interest for studying the Mo—MP—Si (MP = Pt, Ru) system follows from the attractive properties of MoSi_2_ regarding high-temperature oxidation. But early in the past, the first studies performed by Fitzer (1955) mentioned the poor behaviour of this material under oxidizing atmosphere at moderate temperatures (300–600°C) due to a catastrophic degradation, the so-called "pest phenomenon". Consequently, one of the challenges for application of MoSi_2_ is to control the pest oxidation by adding alloying elements (Ru, Pt, B, Al, Ge, Y, Ti, Zr, Ta) (Littner, 2003, Benarchid *et al.* 2009) and/or by controlling the microstructure. Recently, the optimization of the microstructure led to fully densified materials showing dramatically improved oxidation performance of MoSi_2_ (Cabouro *et al.*, 2007, Cabouro *et al.*, 2008, Knittel *et al.*, 2010), Bernard *et al.*, 2010. In the course of our studies focused on the evaluation of the effect of elemental substitutions, we studied the isothermal section of the ternary phase diagram Mo—Pt—Si at 1423 K. Two new phases of composition MoPt_2_Si_3_ and MoPt_3_Si_4_ were identified (Littner, 2003), and MoPt_3_Si_4_ was indexed in the orthorhombic system: a = 5.5096 Å, b = 3.493 Å, c = 24.294 Å. The structure of MoPt_3_Si_4_ was recently published by Joubert *et al.* (2010), from powder X-ray diffraction data. It turned out that the MoPt_3_Si_4_ structure can be derived from PtSi by an ordered substitution of Pt by Mo. The atomic arrangement along the *c* axis leads to a fourfold superstructure, with *c*(MoPt_3_Si_4_) = 4 × *c*(PtSi). Our analysis based on single-crystal data confirms the previous results but yields more accurate atomic positions, approximately by one order of magnitude. Additionally, this also allows determination of anisotropic displacement parameters (Fig. 1). Fig. 2 shows the coordination polyhedra. The coordination number for each d metal is 10, for Si it is 8. Mo and Pt exhibit different coordination polyhedra. In both cases, these are based on bicapped-square antiprisms, but with two additional vertices in *cis*-positions for Mo whereas they are in *trans*-positions for Pt (as in PtSi). The coordination polyhedra for Si1, Si2 and Si3 atoms can be considered as highly deformed square antiprisms (as in PtSi), while Si4 has a bicapped trigonal prismatic coordination geometry. Shortest and longest interatomic distances for each coordination polyedra are reported in Table 1. The shortest distances found for Mo—Si and Pt—Si are 2.535 (2) Å and of 2.387 (3) Å respectively. These may be compared to the values given by Joubert *et al.* (2010), namely 2.463 Å and 2.355 Å.

Figure 3 also emphasizes that the corrugated ribbons formed by the silicon sub-network and expanded along the *c* axis are greatly distorted compared to the PtSi parent structure.

### Experimental

Metal powders with nominal purities > 99.9 (Pt sponge 270 mesh - Engelhard - Clal, Si and Mo 325 mesh: Cerac) were mixed in different atomic ratios corresponding to alloys belonging to the MoSi_2_—Mo_5_Si_3_—PtSi domain. An ingot was prepared by high frequency melting, and stabilized in a thermodynamic equilibrium for 100 h at 1150°C under argon. Single crystals of Pt_3_MoSi_4_ were directly isolated from the crushed ingot. A part of the ingot was embedded in an epoxy resin, polished and microanalyzed by an electron probe (SX 50 CAMECA, - PAP correction program (Pouchou & Pichoir, 1984). The EPMA composition corresponds, within the accuracy of the measurement, to that obtained by the structural determination.

### Refinement

Maximum residual electron density: highest peak 2.16 found at 0.75 Å from Pt2 and minimum residual electron density: highest hole -3.81 found at 1.22 Å from Si4

### Crystal data

**Table:**

MoPt_3_Si_4_	*D*_x_ = 11.256 Mg m^−^^3^
*M**_r_* = 793.57	Melting point: 1503 K
Orthorhombic, *P**n**m**a*	Mo *K*α radiation, λ = 0.71073 Å
Hall symbol: -P 2ac 2n	Cell parameters from 4246 reflections
*a* = 5.5121 (2) Å	θ = 3.4–33.7°
*b* = 3.4951 (1) Å	µ = 92.80 mm^−^^1^
*c* = 24.3078 (7) Å	*T* = 297 K
*V* = 468.30 (3) Å^3^	Needle, metallic colourless
*Z* = 4	0.12 × 0.03 × 0.03 mm
*F*(000) = 1328	

### Data collection

**Table:**

Bruker APEXII QUAZAR CCD diffractometer	1060 independent reflections
Radiation source: ImuS	955 reflections with *I* > 2σ(*I*)
graphite	*R*_int_ = 0.040
ω scans	θ_max_ = 33.7°, θ_min_ = 1.7°
Absorption correction: multi-scan (*SADABS*; Bruker, 2004)	*h* = −8→8
*T*_min_ = 0.031, *T*_max_ = 0.174	*k* = −5→5
9779 measured reflections	*l* = −34→37

### Refinement

**Table:**

Refinement on *F*^2^	Primary atom site location: structure-invariant direct methods
Least-squares matrix: full	Secondary atom site location: difference Fourier map
*R*[*F*^2^ > 2σ(*F*^2^)] = 0.029	*w* = 1/[σ^2^(*F*_o_^2^) + (0.0069*P*)^2^ + 14.4673*P*] where *P* = (*F*_o_^2^ + 2*F*_c_^2^)/3
*wR*(*F*^2^) = 0.067	(Δ/σ)_max_ = 0.001
*S* = 1.41	Δρ_max_ = 2.16 e Å^−^^3^
1060 reflections	Δρ_min_ = −3.81 e Å^−^^3^
50 parameters	Extinction correction: *SHELXL97* (Sheldrick, 2008), Fc^*^=kFc[1+0.001xFc^2^λ^3^/sin(2θ)]^-1/4^
0 restraints	Extinction coefficient: 0.00129 (7)

### Special details

**Table:**

Geometry. All e.s.d.'s (except the e.s.d. in the dihedral angle between two l.s. planes) are estimated using the full covariance matrix. The cell e.s.d.'s are taken into account individually in the estimation of e.s.d.'s in distances, angles and torsion angles; correlations between e.s.d.'s in cell parameters are only used when they are defined by crystal symmetry. An approximate (isotropic) treatment of cell e.s.d.'s is used for estimating e.s.d.'s involving l.s. planes.
Refinement. Refinement of *F*^2^ against ALL reflections. The weighted *R*-factor *wR* and goodness of fit *S* are based on *F*^2^, conventional *R*-factors *R* are based on *F*, with *F* set to zero for negative *F*^2^. The threshold expression of *F*^2^ > σ(*F*^2^) is used only for calculating *R*-factors(gt) *etc*. and is not relevant to the choice of reflections for refinement. *R*-factors based on *F*^2^ are statistically about twice as large as those based on *F*, and *R*- factors based on ALL data will be even larger.

### Fractional atomic coordinates and isotropic or equivalent isotropic displacement parameters (Å^2^)

**Table:**

	*x*	*y*	*z*	*U*_iso_*/*U*_eq_	
Pt1	0.50285 (7)	0.2500	0.547907 (16)	0.00783 (11)	
Pt2	0.00468 (7)	−0.2500	0.669289 (16)	0.00688 (11)	
Pt3	0.00210 (7)	0.2500	0.574730 (17)	0.00783 (11)	
Mo1	−0.49497 (15)	−0.2500	0.71035 (4)	0.00562 (16)	
Si1	0.6616 (6)	−0.2500	0.60935 (12)	0.0071 (5)	
Si2	0.3216 (6)	0.2500	0.64573 (12)	0.0072 (5)	
Si3	0.1790 (6)	−0.2500	0.51984 (12)	0.0076 (5)	
Si4	−0.1678 (6)	−0.7500	0.72502 (12)	0.0071 (5)	

### Atomic displacement parameters (Å^2^)

**Table:**

	*U*^11^	*U*^22^	*U*^33^	*U*^12^	*U*^13^	*U*^23^
Pt1	0.00828 (19)	0.00729 (18)	0.00793 (19)	0.000	0.00011 (13)	0.000
Pt2	0.00732 (18)	0.00574 (16)	0.00759 (19)	0.000	0.00034 (13)	0.000
Pt3	0.00835 (18)	0.00728 (17)	0.00787 (18)	0.000	−0.00006 (13)	0.000
Mo1	0.0068 (3)	0.0044 (3)	0.0057 (3)	0.000	0.0003 (3)	0.000
Si1	0.0086 (11)	0.0063 (11)	0.0064 (12)	0.000	−0.0006 (10)	0.000
Si2	0.0092 (12)	0.0055 (12)	0.0068 (12)	0.000	−0.0021 (10)	0.000
Si3	0.0090 (12)	0.0063 (12)	0.0075 (11)	0.000	0.0004 (10)	0.000
Si4	0.0099 (12)	0.0033 (11)	0.0082 (12)	0.000	−0.0006 (10)	0.000

### Geometric parameters (Å, °)

**Table:**

Pt1—Si3^i^	2.406 (3)	Mo1—Si1^v^	2.602 (3)
Pt1—Si1	2.460 (2)	Mo1—Pt2^xi^	2.9256 (10)
Pt1—Si1^ii^	2.460 (2)	Mo1—Pt2	2.929 (1)
Pt1—Si2	2.579 (3)	Mo1—Pt2^v^	2.9331 (10)
Pt1—Si3	2.590 (2)	Si1—Pt2^iii^	2.387 (3)
Pt1—Si3^ii^	2.590 (2)	Si1—Pt1^vi^	2.460 (2)
Pt1—Pt3^iii^	2.8281 (6)	Si1—Pt1	2.460 (2)
Pt1—Pt3	2.8362 (6)	Si1—Mo1^iii^	2.602 (3)
Pt1—Pt1^i^	2.9119 (6)	Si1—Pt3^iii^	2.699 (2)
Pt1—Pt1^iv^	2.9119 (6)	Si1—Pt3^xiii^	2.699 (2)
Pt2—Si1^v^	2.387 (3)	Si1—Si2^vi^	2.711 (3)
Pt2—Si4^ii^	2.407 (2)	Si1—Si2	2.711 (3)
Pt2—Si4	2.407 (2)	Si2—Pt3	2.466 (3)
Pt2—Si2	2.536 (2)	Si2—Pt2^ii^	2.536 (2)
Pt2—Si2^vi^	2.536 (2)	Si2—Pt2	2.536 (2)
Pt2—Pt3^vi^	2.8874 (5)	Si2—Mo1^xiv^	2.558 (2)
Pt2—Pt3	2.8874 (5)	Si2—Mo1^iii^	2.558 (2)
Pt2—Mo1^vii^	2.9255 (10)	Si2—Pt1	2.579 (3)
Pt2—Mo1	2.9294 (10)	Si2—Si1^ii^	2.711 (3)
Pt2—Mo1^iii^	2.9331 (10)	Si2—Si1	2.711 (3)
Pt3—Si3	2.405 (2)	Si3—Pt1^vi^	2.590 (2)
Pt3—Si3^ii^	2.405 (2)	Si3—Pt1	2.590 (2)
Pt3—Si2	2.466 (3)	SI3—Pt3	2.405 (2)
Pt3—Si3^viii^	2.506 (3)	Si3—Pt3^vi^	2.405 (2)
Pt3—Si1^v^	2.699 (2)	Si3—Pt1^i^	2.406 (3)
Pt3—Si1^ix^	2.699 (2)	Si3—Pt3^viii^	2.506 (3)
Pt3—Pt1^v^	2.8281 (6)	Si3—Si3^xv^	2.807 (5)
Pt3—Pt1	2.836 (1)	Si3—Si3^viii^	2.807 (5)
Pt3—Pt2^ii^	2.8874 (5)	Si4—Pt2^vi^	2.407 (2)
Pt3—Pt2	2.8874 (5)	Si4—Pt2	2.407 (2)
Mo1—Si4	2.535 (2)	Si4—Mo1^vii^	2.535 (2)
Mo1—Si4^ii^	2.537 (2)	Si4—Mo1^xvi^	2.535 (2)
Mo1—Si4^x^	2.535 (2)	Si4—Mo1^vi^	2.537 (2)
Mo1—Si4^xi^	2.535 (2)	Si4—Mo1	2.536 (2)
Mo1—Si2^xii^	2.558 (2)	Si4—Si4^vii^	3.012 (5)
Mo1—Si2^v^	2.558 (2)	Si4—Si4^xi^	3.012 (5)
			
Si3^i^—Pt1—Si1	98.99 (9)	Si4^ii^—Mo1—Si4	87.10 (10)
Si3^i^—Pt1—Si1^ii^	98.99 (9)	Si4^x^—Mo1—Si2^xii^	134.65 (10)
Si1—Pt1—Si1^ii^	90.54 (10)	Si4^xi^—Mo1—Si2^xii^	76.18 (8)
Si3^i^—Pt1—Si2	155.99 (10)	Si4^ii^—Mo1—Si2^xii^	146.93 (10)
Si1—Pt1—Si2	65.04 (8)	Si4—Mo1—Si2^xii^	84.07 (8)
Si1^ii^—Pt1—Si2	65.04 (8)	Si4^x^—Mo1—Si2^v^	76.18 (8)
Si3^i^—Pt1—Si3	108.80 (8)	Si4^xi^—Mo1—Si2^v^	134.65 (10)
Si1—Pt1—Si3	85.75 (8)	Si4^ii^—Mo1—Si2^v^	84.07 (8)
Si1^ii^—Pt1—Si3	152.21 (10)	Si4—Mo1—Si2^v^	146.93 (10)
Si2—Pt1—Si3	88.61 (8)	Si2^xii^—Mo1—Si2^v^	86.18 (9)
Si3^i^—Pt1—Si3^ii^	108.80 (8)	Si4^x^—Mo1—Si1^v^	135.16 (5)
Si1—Pt1—Si3^ii^	152.21 (10)	Si4^xi^—Mo1—Si1^v^	135.16 (5)
Si1^ii^—Pt1—Si3^ii^	85.75 (8)	Si4^ii^—Mo1—Si1^v^	84.07 (9)
Si2—Pt1—Si3^ii^	88.61 (8)	Si4—Mo1—Si1^v^	84.08 (9)
Si3—Pt1—Si3^ii^	84.88 (9)	Si2^xii^—Mo1—Si1^v^	63.37 (8)
Si3^i^—Pt1—Pt3^iii^	56.52 (7)	Si2^v^—Mo1—Si1^v^	63.37 (8)
Si1—Pt1—Pt3^iii^	60.91 (7)	Si4^x^—Mo1—Pt2^xi^	51.70 (6)
Si1^ii^—Pt1—Pt3^iii^	60.91 (7)	Si4^xi^—Mo1—Pt2^xi^	51.70 (6)
Si2—Pt1—Pt3^iii^	99.47 (7)	Si4^ii^—Mo1—Pt2^xi^	81.95 (7)
Si3—Pt1—Pt3^iii^	137.02 (5)	Si4—Mo1—Pt2^xi^	81.94 (7)
Si3^ii^—Pt1—Pt3^iii^	137.02 (5)	Si2^xii^—Mo1—Pt2^xi^	127.87 (6)
Si3^i^—Pt1—Pt3	150.09 (8)	Si2^v^—Mo1—Pt2^xi^	127.87 (6)
Si1—Pt1—Pt3	101.93 (7)	Si1^v^—Mo1—Pt2^xi^	160.67 (8)
Si1^ii^—Pt1—Pt3	101.93 (7)	Si4^x^—Mo1—Pt2	124.35 (7)
Si2—Pt1—Pt3	53.91 (7)	Si4^xi^—Mo1—Pt2	124.35 (7)
Si3—Pt1—Pt3	52.39 (6)	Si4^ii^—Mo1—Pt2	51.64 (6)
Si3^ii^—Pt1—Pt3	52.39 (6)	Si4—Mo1—Pt2	51.64 (6)
Pt3^iii^—Pt1—Pt3	153.38 (2)	Si2^xii^—Mo1—Pt2	99.35 (7)
Si3^i^—Pt1—Pt1^i^	57.34 (5)	Si2^v^—Mo1—Pt2	99.35 (7)
Si1—Pt1—Pt1^i^	93.62 (5)	Si1^v^—Mo1—Pt2	50.71 (7)
Si1^ii^—Pt1—Pt1^i^	156.33 (7)	Pt2^xi^—Mo1—Pt2	109.96 (3)
Si2—Pt1—Pt1^i^	137.15 (4)	Si4^x^—Mo1—Pt2^v^	81.81 (7)
Si3—Pt1—Pt1^i^	51.45 (7)	Si4^xi^—Mo1—Pt2^v^	81.81 (7)
Si3^ii^—Pt1—Pt1^i^	100.77 (6)	Si4^ii^—Mo1—Pt2^v^	135.76 (5)
Pt3^iii^—Pt1—Pt1^i^	101.239 (19)	Si4—Mo1—Pt2^v^	135.76 (5)
Pt3—Pt1—Pt1^i^	99.983 (19)	Si2^xii^—Mo1—Pt2^v^	54.50 (6)
Si3^i^—Pt1—Pt1^iv^	57.34 (5)	Si2^v^—Mo1—Pt2^v^	54.50 (6)
Si1—Pt1—Pt1^iv^	156.33 (7)	Si1^v^—Mo1—Pt2^v^	89.47 (7)
Si1^ii^—Pt1—Pt1^iv^	93.62 (5)	Pt2^xi^—Mo1—Pt2^v^	109.86 (3)
Si2—Pt1—Pt1^iv^	137.15 (4)	Pt2—Mo1—Pt2^v^	140.18 (4)
Si3—Pt1—Pt1^iv^	100.77 (6)	Pt2^iii^—Si1—Pt1	130.73 (7)
Si3^ii^—Pt1—Pt1^iv^	51.45 (7)	Pt2^iii^—Si1—Pt1^vi^	130.73 (7)
Pt3^iii^—Pt1—Pt1^iv^	101.239 (19)	Pt1—Si1—Pt1^vi^	90.54 (10)
Pt3—Pt1—Pt1^iv^	99.983 (19)	Pt2^iii^—Si1—Mo1^iii^	71.76 (8)
Pt1^i^—Pt1—Pt1^iv^	73.760 (19)	Pt1—Si1—Mo1^iii^	117.05 (9)
Si1^v^—Pt2—Si4^ii^	91.76 (9)	Pt1^vi^—Si1—Mo1^iii^	117.05 (9)
Si1^v^—Pt2—Si4	91.76 (9)	Pt2^iii^—Si1—Pt3^iii^	68.87 (7)
Si4^ii^—Pt2—Si4	93.12 (11)	Pt1—Si1—Pt3^iii^	66.30 (4)
Si1^v^—Pt2—Si2	114.06 (8)	Pt1^vi^—Si1—Pt3^iii^	121.20 (11)
Si4^ii^—Pt2—Si2	84.19 (8)	Mo1^iii^—Si1—Pt3^iii^	121.65 (8)
Si4—Pt2—Si2	154.07 (10)	Pt2^iii^—Si1—Pt3^xiii^	68.87 (7)
Si1^v^—Pt2—Si2^vi^	114.06 (8)	Pt1—Si1—Pt3^xiii^	121.20 (11)
Si4^ii^—Pt2—Si2^vi^	154.07 (10)	Pt1^vi^—Si1—Pt3^xiii^	66.30 (4)
Si4—Pt2—Si2^vi^	84.19 (8)	Mo1^iii^—Si1—Pt3^xiii^	121.65 (8)
Si2—Pt2—Si2^vi^	87.10 (10)	Pt3^iii^—Si1—Pt3^xiii^	80.71 (9)
Si1^v^—Pt2—Pt3^vi^	60.68 (5)	Pt2^iii^—Si1—Si2	110.40 (11)
Si4^ii^—Pt2—Pt3^vi^	152.32 (7)	Pt1—Si1—Si2	59.61 (7)
Si4—Pt2—Pt3^vi^	90.38 (6)	Pt1^vi^—Si1—Si2	114.21 (13)
Si2—Pt2—Pt3^vi^	103.92 (6)	Mo1^iii^—Si1—Si2	57.52 (8)
Si2^vi^—Pt2—Pt3^vi^	53.60 (6)	Pt3^iii^—Si1—Si2	99.50 (4)
Si1^v^—Pt2—Pt3	60.68 (5)	Pt3^xiii^—Si1—Si2	179.13 (14)
Si4^ii^—Pt2—Pt3	90.38 (6)	Pt2^iii^—Si1—Si2^vi^	110.40 (11)
Si4—Pt2—Pt3	152.32 (7)	Pt1—Si1—Si2^vi^	114.21 (13)
Si2—Pt2—Pt3	53.60 (6)	Pt1^vi^—Si1—Si2^vi^	59.61 (7)
Si2^vi^—Pt2—Pt3	103.93 (6)	Mo1^iii^—Si1—Si2^vi^	57.52 (8)
Pt3^vi^—Pt2—Pt3	74.490 (14)	Pt3^iii^—Si1—Si2^vi^	179.13 (14)
Si1^v^—Pt2—Mo1^vii^	127.65 (7)	Pt3^xiii^—Si1—Si2^vi^	99.50 (4)
Si4^ii^—Pt2—Mo1^vii^	55.76 (6)	Si2—Si1—Si2^vi^	80.27 (12)
Si4—Pt2—Mo1^vii^	55.76 (6)	Pt3—Si2—Pt2^ii^	70.50 (7)
Si2—Pt2—Mo1^vii^	103.02 (7)	Pt3—Si2—Pt2	70.50 (7)
Si2^vi^—Pt2—Mo1^vii^	103.02 (7)	Pt2^ii^—Si2—Pt2	87.10 (10)
Pt3^vi^—Pt2—Mo1^vii^	142.754 (7)	Pt3—Si2—Mo1^xiv^	135.41 (6)
Pt3—Pt2—Mo1^vii^	142.754 (7)	Pt2^ii^—Si2—Mo1^xiv^	70.30 (5)
Si1^v^—Pt2—Mo1	57.54 (7)	Pt2—Si2—Mo1^xiv^	127.17 (12)
Si4^ii^—Pt2—Mo1	55.73 (7)	Pt3—Si2—Mo1^iii^	135.41 (6)
Si4—Pt2—Mo1	55.73 (7)	Pt2^ii^—Si2—Mo1^iii^	127.17 (12)
Si2—Pt2—Mo1	136.42 (5)	Pt2—Si2—Mo1^iii^	70.30 (5)
Si2^vi^—Pt2—Mo1	136.42 (5)	Mo1^xiv^—Si2—Mo1^iii^	86.17 (9)
Pt3^vi^—Pt2—Mo1	105.463 (19)	Pt3—Si2—Pt1	68.37 (8)
Pt3—Pt2—Mo1	105.463 (19)	Pt2^ii^—Si2—Pt1	118.36 (8)
Mo1^vii^—Pt2—Mo1	70.12 (2)	Pt2—Si2—Pt1	118.36 (8)
Si1^v^—Pt2—Mo1^iii^	162.29 (8)	Mo1^xiv^—Si2—Pt1	114.39 (9)
Si4^ii^—Pt2—Mo1^iii^	100.36 (8)	Mo1^iii^—Si2—Pt1	114.39 (9)
Si4—Pt2—Mo1^iii^	100.36 (8)	Pt3—Si2—Si1^ii^	105.40 (11)
Si2—Pt2—Mo1^iii^	55.20 (6)	Pt2^ii^—Si2—Si1^ii^	96.07 (4)
Si2^vi^—Pt2—Mo1^iii^	55.20 (6)	Pt2—Si2—Si1^ii^	173.71 (13)
Pt3^vi^—Pt2—Mo1^iii^	105.996 (19)	Mo1^xiv^—Si2—Si1^ii^	59.11 (7)
Pt3—Pt2—Mo1^iii^	105.996 (19)	Mo1^iii^—Si2—Si1^ii^	111.55 (13)
Mo1^vii^—Pt2—Mo1^iii^	70.06 (2)	Pt1—Si2—Si1^ii^	55.35 (8)
Mo1—Pt2—Mo1^iii^	140.18 (4)	Pt3—Si2—Si1	105.40 (11)
Si3—Pt3—Si3^ii^	93.20 (11)	Pt2^ii^—Si2—Si1	173.71 (13)
Si3—Pt3—Si2	95.67 (9)	Pt2—Si2—Si1	96.07 (4)
Si3^ii^—Pt3—Si2	95.67 (9)	Mo1^xiv^—Si2—Si1	111.55 (13)
Si3—Pt3—Si3^viii^	69.67 (9)	Mo1^iii^—Si2—Si1	59.11 (7)
Si3^ii^—Pt3—Si3^viii^	69.67 (9)	Pt1—Si2—Si1	55.35 (8)
Si2—Pt3—Si3^viii^	157.89 (10)	Si1^ii^—Si2—Si1	80.27 (12)
Si3—Pt3—Si1^v^	89.11 (7)	Pt3—Si3—Pt3^vi^	93.20 (11)
Si3^ii^—Pt3—Si1^v^	157.71 (9)	Pt3—Si3—Pt1^i^	132.47 (6)
Si2—Pt3—Si1^v^	106.16 (8)	Pt3^vi^—Si3—Pt1^i^	132.47 (6)
Si3^viii^—Pt3—Si1^v^	90.52 (8)	Pt3—Si3—Pt3^viii^	110.33 (9)
Si3—Pt3—Si1^ix^	157.72 (9)	Pt3^vi^—Si3—Pt3^viii^	110.33 (9)
Si3^ii^—Pt3—Si1^ix^	89.11 (7)	Pt1^i^—Si3—Pt3^viii^	70.27 (8)
Si2—Pt3—Si1^ix^	106.16 (8)	Pt3—Si3—Pt1^vi^	128.58 (13)
Si3^viii^—Pt3—Si1^ix^	90.52 (8)	Pt3^vi^—Si3—Pt1^vi^	69.08 (4)
Si1^v^—Pt3—Si1^ix^	80.71 (9)	Pt1^i^—Si3—Pt1^vi^	71.20 (7)
Si3—Pt3—Pt1^v^	105.46 (7)	Pt3^viii^—Si3—Pt1^vi^	121.08 (9)
Si3^ii^—Pt3—Pt1^v^	105.46 (7)	Pt3—Si3—Pt1	69.08 (4)
Si2—Pt3—Pt1^v^	148.91 (8)	Pt3^vi^—Si3—Pt1	128.58 (13)
Si3^viii^—Pt3—Pt1^v^	53.20 (7)	Pt1^i^—Si3—Pt1	71.20 (7)
Si1^v^—Pt3—Pt1^v^	52.79 (6)	Pt3^viii^—Si3—Pt1	121.08 (9)
Si1^ix^—Pt3—Pt1^v^	52.79 (6)	Pt1^vi^—Si3—Pt1	84.88 (9)
Si3—Pt3—Pt1	58.53 (7)	Pt3—Si3—Si3^xv^	110.98 (16)
Si3^ii^—Pt3—Pt1	58.53 (7)	Pt3^vi^—Si3—Si3^xv^	56.86 (7)
Si2—Pt3—Pt1	57.71 (7)	Pt1^i^—Si3—Si3^xv^	106.10 (14)
Si3^viii^—Pt3—Pt1	100.18 (7)	Pt3^viii^—Si3—Si3^xv^	53.47 (10)
Si1^v^—Pt3—Pt1	138.45 (5)	Pt1^vi^—Si3—Si3^xv^	98.92 (3)
Si1^ix^—Pt3—Pt1	138.45 (5)	Pt1—Si3—Si3^xv^	174.46 (16)
Pt1^v^—Pt3—Pt1	153.38 (2)	Pt3—Si3—Si3^viii^	56.86 (7)
Si3—Pt3—Pt2^ii^	151.56 (8)	Pt3^vi^—Si3—Si3^viii^	110.98 (16)
Si3^ii^—Pt3—Pt2^ii^	89.99 (6)	Pt1^i^—Si3—Si3^viii^	106.10 (14)
Si2—Pt3—Pt2^ii^	55.90 (5)	Pt3^viii^—Si3—Si3^viii^	53.47 (10)
Si3^viii^—Pt3—Pt2^ii^	137.07 (3)	Pt1^vi^—Si3—Si3^viii^	174.46 (16)
Si1^v^—Pt3—Pt2^ii^	98.46 (5)	Pt1—Si3—Si3^viii^	98.92 (3)
Si1^ix^—Pt3—Pt2^ii^	50.46 (6)	Si3^xv^—Si3—Si3^viii^	77.02 (16)
Pt1^v^—Pt3—Pt2^ii^	100.856 (15)	Pt2—Si4—Pt2^vi^	93.11 (11)
Pt1—Pt3—Pt2^ii^	100.265 (15)	Pt2—Si4—Mo1^vii^	72.54 (5)
Si3—Pt3—Pt2	89.99 (6)	Pt2^vi^—Si4—Mo1^vii^	134.49 (14)
Si3^ii^—Pt3—Pt2	151.57 (8)	Pt2—Si4—Mo1^xvi^	134.49 (14)
Si2—Pt3—Pt2	55.90 (5)	Pt2^vi^—Si4—Mo1^xvi^	72.54 (5)
Si3^viii^—Pt3—Pt2	137.07 (3)	Mo1^vii^—Si4—Mo1^xvi^	87.14 (10)
Si1^v^—Pt3—Pt2	50.46 (6)	Pt2—Si4—Mo1^vi^	134.58 (13)
Si1^ix^—Pt3—Pt2	98.46 (5)	Pt2^vi^—Si4—Mo1^vi^	72.63 (4)
Pt1^v^—Pt3—Pt2	100.855 (15)	Mo1^vii^—Si4—Mo1^vi^	146.00 (13)
Pt1—Pt3—Pt2	100.265 (15)	Mo1^xvi^—Si4—Mo1^vi^	83.07 (4)
Pt2^ii^—Pt3—Pt2	74.490 (14)	Pt2—Si4—Mo1	72.63 (4)
Si4^x^—Mo1—Si4^xi^	87.14 (10)	Pt2^vi^—Si4—Mo1	134.58 (13)
Si4^x^—Mo1—Si4^ii^	72.85 (5)	Mo1^vii^—Si4—Mo1	83.07 (4)
Si4^xi^—Mo1—Si4^ii^	130.91 (7)	Mo1^xvi^—Si4—Mo1	146.01 (13)
Si4^x^—Mo1—Si4	130.91 (6)	Mo1^vi^—Si4—Mo1	87.10 (10)
Si4^xi^—Mo1—Si4	72.85 (5)		

Symmetry codes: (i) −*x*+1, −*y*, −*z*+1; (ii) *x*, *y*+1, *z*; (iii) *x*+1, *y*, *z*; (iv) −*x*+1, −*y*+1, −*z*+1; (v) *x*−1, *y*, *z*; (vi) *x*, *y*−1, *z*; (vii) *x*+1/2, *y*, −*z*+3/2; (viii) −*x*, −*y*, −*z*+1; (ix) *x*−1, *y*+1, *z*;  (x) *x*−1/2, *y*+1, −*z*+3/2; (xi) *x*−1/2, *y*, −*z*+3/2; (xii) *x*−1, *y*−1, *z*; (xiii) *x*+1, *y*−1, *z*; (xiv) *x*+1, *y*+1, *z*; (xv) −*x*, −*y*−1, −*z*+1; (xvi) *x*+1/2, *y*−1, −*z*+3/2.
